# ﻿New subgeneric names for the most commercially important shrimp genus *Penaeus* Fabricius, 1798 (Crustacea, Decapoda, Penaeidae)

**DOI:** 10.3897/zookeys.1141.97349

**Published:** 2023-01-17

**Authors:** Tin-Yam Chan

**Affiliations:** 1 Institute of Marine Biology and Center of Excellence for the Oceans, National Taiwan Ocean University, Keelung 202301, R.O.C., Taiwan National Taiwan Ocean University Keelung Taiwan

**Keywords:** Key, marine invertebrates, new subgenus, nomenclature, stability, taxonomy

## Abstract

Although a recent comprehensive molecular phylogenetic study on *Penaeus* Fabricius, 1798 reinstated a single genus for these economically important shrimps, several clades in the molecular phylogenetic tree do not have formal names. Subgeneric names are given herein to five of these clades if *Penaeus* is to be split. A key to the subgenera in *Penaeus* is also provided.

## ﻿Introduction

The most comprehensive study to date on the phylogenetic relationships amongst the members of the genus *Penaeus* s.l. Fabricius, 1798 was by Yang et al. (2023), which suggested that a single genus should be reinstated for these commercially important shrimps. Their study also proposes that if those molecular clades revealed in the phylogenetic tree of *Penaeus* s.l. (Yang et al. 2023: fig. 3) are recognized as taxonomic groups, the use of subgenera is preferable; the use of this rank would also reduce confusion and maintain stability for non-taxonomists who use the name.

In their phylogenetic study, Yang et al. (2023: fig. 3) showed that up to 11 subgeneric-level clades can be recognized. While many of these clades have been named in the past, five of them, however, remain un-named. In the interest of nomenclatural stability and consistency in discussing their systematics, I here propose to apply formal names for them. This action is justified especially if the peculiar taxon *Marsupenaeus* Tirmizi, 1971, which has a very specialized pouch-like thelycum, is to be maintained.

A key to these 11 subgenera is also provided even though all important characters used have already been proved to be neither synapomorphic nor evolutionary informative in Yang et al. (2023).

## ﻿Systematic account

### ﻿ Penaeus (Penaeus)

Taxon classificationAnimaliaDecapodaPenaeidae

Fabricius, 1798

B953EC4E-6D6A-5B65-9784-187AE1CFF841

#### Type species.

*Penaeusmonodon* Fabricius, 1798.

#### Gender of subgenus.

Masculine.

#### Diagnosis.

Rostrum generally armed with3 ventral teeth. Median sulcus on postrostral carina shallow to indistinct. Adrostral sulcus extending posteriorly more or less to level of epigastric tooth. Gastrofrontal carina absent. Cervical carina with dorsal end a distance from dorsal carapace. Hepatic carina distinct, nearly horizontal. First pereiopod with distinct ischial spine. Fifth pereiopod without exopod. Sixth abdominal somite completely lacking dorsolateral sulcus. Telson without lateral spines. Thelycum closed.

#### Species included.

Penaeus (Penaeus) monodon Fabricius, 1798, Penaeus (Penaeus) simplex Chan, Muchlisin & Hurzaid, 2021.

#### Remarks.

Although this is the nominotypical subgenus of *Penaeus*, it is unusual in lacking an epipod on the fifth pereiopod; the subgenus contains only two of the 32 recognized species in the genus.

### ﻿ Penaeus (Melicertus)

Taxon classificationAnimaliaDecapodaPenaeidae

Rafinesque, 1814

D4502D51-D502-54EC-AC36-1E26F63020E1

#### Type species.

*Melicertustigrinus* Rafinesque, 1814 (= *Cancerkerathurus* Forskål, 1775).

#### Gender of subgenus.

Masculine.

#### Diagnosis.

Rostrum usually bearing 1 ventral tooth. Median sulcus at postrostral carina deep, long, about half carapace length. Adrostral sulcus as wide as postrostral carina, extending to near posterior margin of carapace. Gastrofrontal carina distinct and with posterior end turning anterodorally. Cervical carina long, extending almost to dorsal carapace. Hepatic carina distinct. First pereiopod with ischial spine small to absent. Fifth pereiopod bearing exopod. Sixth abdominal somite completely lacking dorsolateral sulcus. Telson with 3 pairs of lateral spines. Thelycum closed.

#### Species included.

Penaeus (Melicertus) kerathurus (Forskål, 1775).

#### Remarks.

Amongst the members of *Penaeus*, only this subgenus has a geographical distribution in the eastern Atlantic and the Mediterranean. This subgenus is also unique in the genus by having a long cervical carina which has the dorsal end almost reaching the dorsal carapace.

### ﻿ Penaeus (Fenneropenaeus)

Taxon classificationAnimaliaDecapodaPenaeidae

Pérez Farfante, 1969

8703B409-6693-5D04-BF02-510E7C6F55FA

#### Type species.

*Penaeusindicus* H. Milne Edwards, 1837.

#### Gender of subgenus.

Masculine.

#### Diagnosis.

Rostrum generally bearing 2–5 ventral teeth. Postrostral carina without median sulcus, sometimes with pits or sunken areas. Adrostral sulcus extending posteriorly more or less to epigastric tooth. Gastrofrontal carina absent. Cervical carina with dorsal end a distance from dorsal carapace. Hepatic carina often absent, if present, ill-defined. First pereiopod with small to minute ischial spine. Fifth pereiopod bearing exopod. Sixth abdominal somite completely lacking dorsolateral sulcus. Telson without lateral spines. Thelycum closed.

#### Species included.

Penaeus (Fenneropenaeus) chinensis (Osbeck, 1765), Penaeus (Fenneropenaeus) indicus H. Milne Edwards, 1837, Penaeus (Fenneropenaeus) merguiensis De Man, 1888, Penaeus (Fenneropenaeus) penicillatus Alcock, 1905, Penaeus (Fenneropenaeus) silasi Muthu & Motoh, 1979.

#### Remarks.

This subgenus is unique in the genus by lacking a distinct hepatic carina. Only P. (Fenneropenaeus) chinensis bears an ill-defined hepatic carina while all other species of Penaeus (Fenneropenaeus) lack a hepatic carina. As mentioned in Ma et al. (2011) and Yang et al. (2023), *Fenneropenaeuskonkani* Chanda & Bhattacharya, 2003 is very likely to be an invalid taxon with a deformed rostrum and a synonym of a known species of Penaeus (Fenneropenaeus).

### ﻿ Penaeus (Litopenaeus)

Taxon classificationAnimaliaDecapodaPenaeidae

Pérez Farfante, 1969

AD209BB4-B8A3-5E97-8FC9-6CEB424DEA54

#### Type species.

*Penaeusvannamei* Boone, 1931.

#### Gender of subgenus.

Masculine.

#### Diagnosis.

Rostrum usually bearing 2–4 ventral teeth. Postrostral carina without median sulcus, only sometimes with pits or sunken areas. Adrostral sulcus extending posteriorly more or less to epigastric tooth. Gastrofrontal carina absent. Cervical carina with dorsal end a distance from dorsal carapace. Hepatic carina distinct. First pereiopod with distinct ischial spine. Fifth pereiopod bearing exopod. Sixth abdominal somite bearing weak to distinct dorsolateral sulcus. Telson without lateral spines. Thelycum open.

#### Species included.

Penaeus (Litopenaeus) occidentalis Streets, 1871; Penaeus (Litopenaeus) schmitti Burkenroad, 1936; Penaeus (Litopenaeus) setiferus (Linnaeus, 1767); Penaeus (Litopenaeus) stylirostris Stimpson, 1871; Penaeus (Litopenaeus) vannamei Boone, 1931.

#### Remarks.

This subgenus is unique in the genus by having an open thelycum.

### ﻿ Penaeus (Marsupenaeus)

Taxon classificationAnimaliaDecapodaPenaeidae

Tirmizi, 1971

AF32655F-428F-5844-B2BD-F20E7DCAF5AE

#### Type species.

Penaeuscanaliculatusvar.japonicus Bate, 1888.

#### Gender of subgenus.

Masculine.

#### Diagnosis.

Rostrum generally armed with1 ventral tooth. Median sulcus at postrostral carina deep, long, about half carapace length. Adrostral sulcus extending to near posterior margin of carapace, posterior part somewhat narrower than postrostal carina. Gastrofrontal carina distinct, with posterior end turning anterodorsally. Cervical carina with dorsal end a distance from dorsal carapace. Hepatic carina distinct. First pereiopod with ischial spine minute or absent. Fifth pereiopod bearing exopod. Sixth abdominal somite completely lacking dorsolateral sulcus. Telson with 3 pairs of lateral spines. Thelycum pouch-like.

#### Species included.

Penaeus (Marsupenaeus) japonicus Bate, 1888, Penaeus (Marsupenaeus) pulchricaudatus Stebbing, 1914.

#### Remarks.

Although this subgenus is unique in the genus by having a highly specialized pouch-like thelycum, males and juveniles are morphologically very similar to those of the subgenus Penaeus (Oleopenaeus) subgen. nov., except for coloration [see “Remarks” under Penaeus (Oleopenaeus) subgen. nov.].

### ﻿ Penaeus (Farfantepenaeus)

Taxon classificationAnimaliaDecapodaPenaeidae

Burukovsky, 1972

0C043511-C290-5F70-AF6F-4E2C5549A367

#### Type species.

Penaeusbrasiliensisvar.aztecus Ives, 1891.

#### Gender of subgenus.

Masculine.

#### Diagnosis.

Rostrum usually bearing 2 ventral teeth. Median sulcus at postrostral carina generally distinct, long. Adrostral sulcus extending to near posterior margin of carapace. Gastrofrontal carina distinct, with posterior end not turning anteriorly. Cervical carina with dorsal end a distance from dorsal carapace. Hepatic carina distinct. First pereiopod with strong ischial spine. Fifth pereiopod bearing exopod. Sixth abdominal somite with distinct dorsolateral sulcus. Telson without lateral spines. Thelycum closed.

#### Species included.

Penaeus (Farfantepenaeus) aztecus Ives, 1891, Penaeus (Farfantepenaeus) brasiensis Latreille, 1817, Penaeus (Farfantepenaeus) brevirostris Kingsley, 1878, Penaeus (Farfantepenaeus) californiensis Holmes, 1900, Penaeus (Farfantepenaeus) duorarum Burkenroad, 1939, Penaeus (Farfantepenaeus) isabelae Tavares & Gusmão, 2016, Penaeus (Farfantepenaeus) notialis Pérez Farfante, 1967, Penaeus (Farfantepenaeus) paulensis Pérez Farfante, 1967, Penaeus (Farfantepenaeus) subtilis Pérez Farfante, 1967.

#### Remarks.

This subgenus together with Penaeus (Litopenaeus) are often called the American *Penaeus*. Morphologically these two subgenera are markedly different from each other and had long been thought to be evolutionary far apart (see Burkenroad 1934; Kubo 1949; Pérez Farfante 1969; Dall et al. 1990; von Sternberg and Motoh 1995; Pérez Farfante and Kensley 1997; von Sternberg 1997). They are, however, very closely related genetically (see Yang et al. 2023). At present only one morphological character, the sixth abdominal somite with dorsolateral sulcus, is found to separate the American *Penaeus* from other congeneric species. Recent molecular analysis has suggested that P. (Farfantepenaeus) notialis, originally described as a subspecies of P. (Farfantepenaeus) duorarum, may not be distinct at the species level (Timm et al. 2019).

### ﻿ Penaeus (Altiopeneaus) subgen. nov.

Taxon classificationAnimaliaDecapodaPenaeidae

3D1CD758-A19C-5E27-87DD-C6438424F7A4

https://zoobank.org/594460D1-98BB-4C4B-9902-068EB4E1DEBA

#### Type species.

*Penaeusmarginatus* Randall, 1840.

#### Gender of subgenus.

Masculine.

#### Diagnosis.

Rostrum usually armed with 2 ventral teeth. Postrostral carina lacking median sulcus. Adrostral sulcus as wide as postrostal carina, extending to near posterior margin of carapace. Gastrofrontal carina distinct, with posterior end turning anterodorsally. Cervical carina with dorsal end a distance from dorsal carapace. Hepatic carina distinct. First pereiopod with strong ischial spine. Fifth pereiopod bearing exopod. Sixth abdominal somite completely lacking dorsolateral sulcus. Telson with 3 pairs of lateral spines. Thelycum closed.

#### Etymology.

The name *Altiopeneaus* (from the Latin *altio* for deeper) alluding to members of this subgenus which have a deeper vertical (depth) distribution than other *Penaeus*.

#### Species included.

Penaeus (Altiopeneaus) marginatus Randall, 1840

#### Remarks.

This taxon corresponds to “gen. nov. 5” in the 11-genus scheme of fig. 3 in Yang et al. (2023). This subgenus is unusual in the genus in inhabiting deeper waters (see Chan 1998). It is also unique amongst the “grooved” species by completely lacking a median sulcus on the postrostral carina.

### ﻿ Penaeus (Eopenaeus) subgen. nov.

Taxon classificationAnimaliaDecapodaPenaeidae

23FC7524-1CF8-5F94-AAE0-1BDE12D6DA3F

https://zoobank.org/4BBD630C-8CB8-4AEE-89F0-949C019DFFAB

#### Type species.

*Penaeussemisulcatus* De Haan, 1844.

#### Gender of subgenus.

Masculine.

#### Diagnosis.

Rostrum generally bearing 3 or 4 ventral teeth. Median sulcus on postrostral carina present or absent. Adrostral sulcus extending posteriorly more or less to level of epigastric tooth. Gastrofrontal carina absent. Cervical carina with dorsal end a distance from dorsal carapace. Hepatic carina distinct and usually sloping anteroventrally. First pereiopod with distinct ischial spine. Fifth pereiopod bearing exopod. Sixth abdominal somite completely lacking dorsolateral sulcus. Telson without lateral spines. Thelycum closed.

#### Etymology.

The name *Eopenaeus* (from the Greek *Eos* for others) refers to this subgenus being morphologically close to the nominotypical subgenus of *Penaeus* while the molecular data revealed that this subgenus is actually more derived than Penaeus (Penaeus) (Yang et al. 2023).

#### Species included.

Penaeus (Eopenaeus) esculentus Haswell, 1879, Penaeus (Eopenaeus) semisulcatus De Haan, 1844.

#### Remarks.

This taxon corresponds to “gen. nov.1” in the 11-genus scheme of fig. 3 in Yang et al. (2023). Morphologically this subgenus is similar to Penaeus (Litopenaeus). Other than having different types of thelycum, these two subgenera can be distinguished by the body coloration [banded in Penaeus (Eopenaeus) subgen. nov. but not banded in Penaeus (Litopenaeus)] and the development of the dorsolateral sulcus on the sixth abominal somite [weak to distinct in Penaeus (Litopenaeus) but completely absent in Penaeus (Eopenaeus) subgen. nov.]. Pérez Farfante (1969) and Pérez Farfante and Kensley (1997) also pointed out that there are differences in the shape of the petasma between these two subgenera, with the ventral costa reaching or not reaching the distal margin of the lateral lobe in Penaeus (Eopenaeus) subgen. nov. and Penaeus (Litopenaeus), respectively.

### ﻿ Penaeus (Ischiopeneaus) subgen. nov.

Taxon classificationAnimaliaDecapodaPenaeidae

3971025D-1157-5A97-B703-E7D4A52A4D2A

https://zoobank.org/716AD2C7-AEDE-4549-94B3-65378708E2DB

#### Type species.

*Penaeuslongistylus* Kubo, 1943.

#### Gender of subgenus.

Masculine.

#### Diagnosis.

Rostrum generally armed with 1 ventral tooth. Median sulcus at postrostral carina deep but distinctly shorter than half carapace length. Adrostral sulcus somewhat wider than postrostal carina and extending to near posterior margin of carapace. Gastrofrontal carina distinct and with posterior end turning anterodorsally. Cervical carina with dorsal end a distance from dorsal carapace. Hepatic carina distinct. First pereiopod with strong ischial spine. Fifth pereiopod bearing exopod. Sixth abdominal somite completely lacking dorsolateral sulcus. Telson with 3 pairs of lateral spines. Thelycum closed.

#### Etymology.

The name *Ischiopenaeus* alludes to the presence of a strong ischial spine at the first pereiopod in this subgenus of *Penaeus*.

#### Species included.

Penaeus (Ischiopenaeus) longistylus Kubo, 1943

#### Remarks.

This taxon corresponds to “gen. nov. 4” in the 11-genus scheme of fig. 3 in Yang et al. (2023). This subgenus differs from almost all the non-American “grooved” species in the first pereiopod bearing a strong ischial spine (vs. small to absent). Another non-American “grooved” species with a strong ischial spine at the first pereiopod is P. (Altiopeneaus) marginatus, which lacks a median sulcus on the postrostral carina and generally has two ventral rostral teeth. Thus, the enigmatic *Melicertussimilis* Chanda & Bhattacharya, 2002 described from the Andaman Sea likely represents juveniles of P. (Ischiopenaeus) longistylus as its original description and figures (Chanda and Bhattacharya 2002: figs 1, 6) indicated the presence of postrostral sulcus, only one ventral rostral tooth and the first pereiopod bearing a strong ischial spine. The “absence” of lateral spines on the telson in *Melicertussimilis* is likely evidence that Chanda and Bhattacharya’s (2002) material are juveniles (total length including rostrum less than 80 mm) as juveniles of *Penaeus* generally have the lateral spines on the telson rather small and can be easily detached or overlooked.

### ﻿ Penaeus (Oleopenaeus) subgen. nov.

Taxon classificationAnimaliaDecapodaPenaeidae

E0C1348C-D09C-5105-87C1-BF23E94DC580

https://zoobank.org/12C57BB8-B27D-4AD7-B3C3-2AD35708E4EC

#### Type species.

*Penaeuslatisulcatus* Kishinouye, 1896.

#### Gender of subgenus.

Masculine.

#### Diagnosis.

Rostrum generally armed with 1 ventral tooth. Median sulcus at postrostral carina deep, long, about half carapace length. Adrostral sulcus as wide as postrostral carina, extending to near posterior margin of carapace. Gastrofrontal carina distinct, with posterior end turning anterodorsally. Cervical carina with dorsal end a distance from dorsal carapace. Hepatic carina distinct. First pereiopod with ischial spine minute or absent. Fifth pereiopod bearing exopod. Sixth abdominal somite completely lacking dorsolateral sulcus. Telson with 3 pairs of lateral spines. Thelycum closed.

#### Etymology.

The name *Oleopenaeus* (from the Latin *olea* for olive coloured) refers to the more or less uniform greenish-yellow body coloration of this group of *Penaeus* shrimps.

#### Species included.

Penaeus (Oleopenaeus) hathor Burkenroad, 1959, Penaeus (Oleopenaeus) latisulcatus Kishinouye, 1896, Penaeus (Oleopenaeus) plebejus Hess, 1865.

#### Remarks.

This taxon corresponds to “gen. nov. 3” in the 11-genus scheme of fig. 3 in Yang et al. (2023). Except for the shape of the thelycum and body coloration, this subgenus is morphologically very similar to Penaeus (Marsupenaeus) (see Chan1998; Tsoi et al. 2014). The thelycum is of the normal closed type in Penaeus (Oleopenaeus) subgen. nov. but pouch-like in Penaeus (Marsupenaeus). With regards to the colour in life, the body is not banded in Penaeus (Oleopenaeus) subgen. nov. but is covered with thick cross bands in Penaeus (Marsupenaeus). The taxonomic status of P. (O.) hathor is still uncertain if it merely represents a subspecies of P. (O.) latisulcatus or even a synonym of the latter, as both morphological and genetic differences between these two taxa are rather minor (Holthuis 1980; Miquel 1984; Chan 1998; Ma et al. 2011; 0.8% sequence divergence in COIb 512 bp, Yang et al. 2023: table 1).

### ﻿ Penaeus (Plagosopenaeus) subgen. nov.

Taxon classificationAnimaliaDecapodaPenaeidae

0B5AEF66-77BB-514B-A967-DFFB85277618

https://zoobank.org/B5F2E1F8-8B97-402E-AC9A-57B8AFCC43EB

#### Type species.

*Palaemoncanaliculatus* Olivier, 1811.

#### Gender of subgenus.

Masculine.

#### Diagnosis.

Rostrum generally bearing 1 ventral tooth. Median sulcus at postrostral carina deep, long, about half carapace length. Adrostral sulcus as wide as postrostral carina, extending to near posterior margin of carapace. Gastrofrontal carina distinct, posterior end turning anterodorsally. Cervical carina with dorsal end a distance from dorsal carapace. Hepatic carina distinct. First pereiopod with ischial spine minute or absent. Fifth pereiopod bearing exopod. Sixth abdominal somite completely lacking dorsolateral sulcus. Telson without lateral spines. Thelycum closed.

#### Etymology.

The name *Plagosopenaeus* (from the Latin *plagosus* for banded) refers to this subgenus of *Penaeus*, which has a very striking banded body coloration.

#### Species included.

Penaeus (Plagosopenaeus) canaliculatus (Olivier, 1811).

#### Remarks.

This taxon corresponds to “gen. nov. 2” in the 11-genus scheme of fig. 3 in Yang et al. (2023). Mophologically, including coloration, this subgenus is extremely similar to Penaeus (Marsupenaeus) (see Yu and Chan 1986; Chan 1998) and such close affinity is also supported by the molecular data (Yang et al. 2023: figs 2, 3). Penaeus (Plagosopenaeus) subgen. nov. only differs from Penaeus (Marsupenaeus) in lacking lateral spines on the telson (vs. bearing three pairs of lateral spines), the thelycum not pouch-like and the last transverse band on the sixth abdominal somite not interrupted (Chan 1998).

### ﻿Key to subgenera in *Penaeus*

**Table:** 

1	Adrostral sulcus and carina long, reaching near posterior margin of carapace; gastrofrontal carina present	**2**
–	Adrostral sulcus and carina short, extending posteriorly at most to mid-carapace around level of epigastric tooth; gastrofrontal carina absent	**8**
2	Gastrofrontal carina not turning anteriorly at posterior end; sixth abdominal tergite with well-defined dorsolateral sulcus	**Penaeus (Farfantepenaeus) Burukovsky, 1972**
–	Gastrofrontal carina turning anterodorsally at posterior end; sixth abdominal tergite without dorsolateral sulcus	**3**
3	Telson lacking lateral spines	**Penaeus (Plagosopenaeus) subgen. nov.**
–	Telson armed with 3 pairs of movable lateral spines	**4**
4	Postrostral carina without median sulcus; usually 2 ventral rostral teeth	**Penaeus (Altiopeneaus) subgen. nov.**
–	Postrostral carina bearing median sulcus; usually 1 ventral rostral tooth	**5**
5	Median sulcus at postrostral carina distinctly shorter than half carapace length; first pereiopod armed with strong ischial spine	**Penaeus (Ischiopeneaus) subgen. nov.**
–	Median sulcus at postrostral carina more or less as long as half carapace length; first pereiopod with ischial spine minute or absent	**6**
6	Cervical carina with dorsal end almost reaching dorsal carapace	**Penaeus (Melicertus) Rafinesque, 1814**
– Cervical carina with dorsal end a distance from dorsal carapace	**7**
7	Thelycum pouch-like; posterior part of adrostral sulcus somewhat narrower than postrostral carina; body banded	**Penaeus (Marsupenaeus) Tirmizi, 1971**
–	Thelycum closed but not pouch-like; adrostral sulcus as wide as postrostral carina; body not banded	**Penaeus (Oleopenaeus) subgen. nov.**
8	Hepatic carina absent or ill-defined	**Penaeus (Fenneropenaeus) Pérez Farfante, 1969**
–	Hepatic carina distinct	**9**
9	Fifth pereiopod without exopod; hepatic carina nearly horizontal	**Penaeus (Penaeus) Fabricius, 1798**
–	Fifth pereiopod bearing exopod; hepatic carina usually sloping anteroventrally	**10**
10	Thelycum open; dorsolateral sulcus, though sometimes rather weak, present on sixth abdominal somite	**Penaeus (Litopenaeus) Pérez Farfante, 1969**
–	Thelycum closed; dorsolateral sulcus completely absent on sixth abdominal somite	**Penaeus (Eopenaeus) subgen. nov.**

## Supplementary Material

XML Treatment for Penaeus (Penaeus)

XML Treatment for Penaeus (Melicertus)

XML Treatment for Penaeus (Fenneropenaeus)

XML Treatment for Penaeus (Litopenaeus)

XML Treatment for Penaeus (Marsupenaeus)

XML Treatment for Penaeus (Farfantepenaeus)

XML Treatment for Penaeus (Altiopeneaus)

XML Treatment for Penaeus (Eopenaeus)

XML Treatment for Penaeus (Ischiopeneaus)

XML Treatment for Penaeus (Oleopenaeus)

XML Treatment for Penaeus (Plagosopenaeus)

## References

[B1] Alcock A (1905) A revision of the “Genus” *Penaeus*, with diagnoses of some new species and varieties. Annals & Magazine of Natural History 16(7): 508–532. 10.1080/03745480509443078

[B2] Bate CS (1888) Report on the Crustacea Macrura Collected by HMS Challenger During the Years1873–1876, Zoology. Neill and Company, Edinburgh, 942 pp. [pls 1–157]

[B3] Boone L (1931) A collection of anomuran and macruran Crustacea from the Bay of Panama and the fresh waters of the Canal Zone. Bulletin of the American Museum of Natural History 63: 137–189.

[B4] Burkenroad MD (1934) Littoral Penaeidea chiefly from the Bingham Oceanographic Collection, with a revision of *Penaeopsis* and descriptions of two new genera and eleven new American species. Bulletin of the Bingham Oceanographic Collection 4: 1–109.

[B5] Burkenroad MD (1936) A new species of *Penaeus* from the American Atlantic. Anais da Academia Brasileira de Ciências 8: 315–318.

[B6] Burkenroad MD (1939) Further observations on Penaeidae of the northern Gulf of Mexico. Bulletin of the Bingham Oceanographic Collection 6(6): 1–62.

[B7] Burkenroad MD (1959) Decapoda Macrura I. Penaeidae. Mission Robert Ph. Dollfusen Egypte XXV. Mission Robert Ph. Dollfusen Égypt. Résultats Scientifique 3: 67–92.

[B8] Burukovsky RN (1972) Some problems of the systematics and distribution of shrimps of the genus *Penaeus*. Trudy AtlantNIRO, Kaliningrad 2: 3–21. [in Russian]

[B9] Chan TY (1998) Shrimps and prawns. In: Carpenter KE Niem VH (Eds) FAO Species Identification Guide for Fishery Purposes.The Living Marine Resources of the Western Central Pacific. Food and Agriculture Organization of the United Nations, Rome, 851–971.

[B10] Chan TY Muchlisin ZA Hurzaid A (2021) Verification of a pseudocryptic species in the commercially important tiger prawn *Penaeusmonodon* Fabricius, 1798 (Decapoda: Penaeidae) from Aceh Province, Indonesia. Journal of Crustacean Biology 41(1): 1–10. 10.1093/jcbiol/ruaa096

[B11] Chanda A Bhattacharya T (2002) *Melicertussimilis*, a new species of prawn, Decapoda: Penaeidae, from India. Journal of the Bombay Natural History Society 99: 495–498.

[B12] Chanda A Bhattacharya T (2003) *Fenneropenaeuskonkani*, a new species of prawn (Decapoda: Penaeidae) from Indian Coast. Science & Culture 69: 229–230.

[B13] Dall W Hill BJ Rothlisberg NW Staples DJ (1990) The biology of Penaeidae. Advances in Marine Biology 27: 1–484.

[B14] de Haan W (1833–1850) Crustacea. In: von Siebold PF (Ed.) Fauna Japonica sive Descriptio Animalium, quae in Itinere per Japoniam, Jussu et Auspiciis Superiorum, qui Summum in India Batava Imperium Tenent, Suspecto, Annis 1823–1830 Collegit, Notis, Observation ibus et Adumbrationibus Illustravit. Lugduni-Batavorum, 243 pp. [pls A-J, L-Q, 1–55]

[B15] de Man JG (1888) Report on the podophthalmous Crustacea of the Mergui Archipelago, collected for the Trustees of the Indian Museum, Calcutta, by Dr. John Anderson F.R.S., Superintendent of the Museum. Zoological Journal of the Linnean Society 22: 1–305. [pls 1–19] 10.1111/j.1096-3642.1888.tb00032.x

[B16] Fabricius JC (1798) Supplementum Entomologiae Systematicae. Proft et Storch, Hafniae, 572 pp.

[B17] Forskål P (1775) Descriptiones Animalium Avium, Amphibiorum, Piscium, Insectorum, Vermium; quæin Itinere Orientali observavit. Mölleri, Hauniæ, 164 pp. 10.5962/bhl.title.2154

[B18] Haswell WA (1879) On the Australian species of *Penaeus*, in the Macleay Museum, Sydney. Proceedings of the Linnean Society of New South Wales 4: 38–44. 10.5962/bhl.part.22836

[B19] Hess W (1865) Beiträge zur Kenntnis der Decapoden-Krebse Ost-Australiens. Archiv für Naturgeschichte 31: 127–173. [pls 6–7] 10.5962/bhl.part.15862

[B20] Holmes SJ (1900) Synopsis of California stalk-eyed Crustacea. Occasional papers of the California Academy of Sciences 7: 1–262. [pls 1–4] 10.5962/bhl.title.31431

[B21] Holthuis LB (1980) FAO species catalogue. Vol. 1 - Shrimps and prawns of the world. An annotated catalogue of species of interest to fisheries. FAO Fisheries Synopsis 125(1): 1–271. [i–xvii]

[B22] Ives JE (1891) Crustacea from the northern coast of Yucatan, the harbor of Vera Cruz, the west coast of Florida and the Bermuda Islands. Proceedings. Academy of Natural Sciences of Philadelphia 1891: 176–207. [pls 5–6]

[B23] Kingsley JS (1878) Notes on the North American Caridea in the Museum of the Peabody Academy of Science at Salem, Mass. Proceedings. Academy of Natural Sciences of Philadelphia 1878: 89–98.

[B24] Kishinouye K (1896) Note on a Japanese *Penaeus* and its classification. Zoological Magazine 8: 372–374. [in Japanese] [Dobutsugaku Zasshi]

[B25] Kubo I (1943) Diagnosis of a new species of the genus *Penaeus*. Suisan Kenkyusi 38: 200–201.

[B26] Kubo I (1949) Studies on penaeids of Japanese and its adjacent waters. Journal of the Tokyo College of Fisheries 36: 1–467.

[B27] Latreille PA (1817) Pénée. *Penaeus*. Nouveau Dictionnaired’Histoire Naturelle 25: 152–156.

[B28] Linnaeus C (1767) Systema Naturæ per Regna triaNaturæ, Secundum Classes, Ordines, Genera, Species, cum Characteribus, Differentiis, Synonymis, Locis (13^th^ Edn., Vol. 1). Vindobonae, 1327 pp. 10.5962/bhl.title.156772

[B29] Ma KY Chan TY Chu KH (2011) Refuting the six-genus classification of *Penaeus* s.l. (Dendrobranchiata, Penaeidae): A combined analysis of mitochondrial and nuclear genes. Zoologica Scripta 40(5): 498–508. 10.1111/j.1463-6409.2011.00483.x

[B30] Milne Edwards H (1834–1840) Histoire naturelle des Crustacés, comprenant l’anatomie, la physiologie et la classification de ces animaux. Librairie encyclopédique de Roret, Paris, 468 pp. [1–532, 1–638, 1–32.] [pls 1–42] 10.5962/bhl.title.16170

[B31] Miquel JC (1984) Notes on Indo-West Pacific Penaeidae, 4. On the two subspecies of *Penaeuslatisulcatus* Kishinouye. Crustaceana 46(1): 104–107. 10.1163/156854084X00108

[B32] Muthu MS Motoh H (1979) On a new species of *Penaeus* (Crustacea, Decapoda, Penaeidae) from North Borneo. Crustacean Research 9(0): 64–70. 10.18353/rcustacea.9.0_64

[B33] Olivier AG (1811) Suite de l’Introduction à l’Histoire Naturelle des Insectes. Palèmon. In: Olivier AG (Ed.) Encyclopédie Méthodique.Histoire Naturelle. Insectes (Vol. 8). H. Agasse, Imprimeur-Libraire, Paris, 656–670.

[B34] Osbeck P (1765) Reisenach Ostindien und China. Nebst O.Toreens Reise nach Suratte und C.G. EkebergsNachricht von der Landwirthschaft der Chineser. Aus dem Schwedischen übersetzt von J.G. Georgi. Rostock, 552 pp. 10.5962/bhl.title.120128

[B35] Pérez Farfante I (1967) A new species and two new subspecies of shrimp of the genus *Penaeus* from the Western Atlantic. Proceedings of the Biological Society of Washington 80: 83–100.

[B36] Pérez Farfante I (1969) Western Atlantic shrimps of the genus *P.naeus*. Fish Bulletin 67: 461–591.

[B37] Pérez Farfante I Kensley B (1997) Penaeoid and sergestoid shrimps and prawns of the world. Keys and diagnoses for the families and genera. Mémoires du Muséum National d’Histoire naturelle, Paris 175: 1–233.

[B38] Rafinesque CS (1814) Précis des Découvertes et Travaux Somiologiques de Mr. C.S. Rafinesque-Schmaltz entre 1800 et 1814 ou Choix Raisonné de ses Principales Découvertesen Zoologie et en Botanique, pour Servir D’introduction á ses Ouvrages Futurs. Royale Typographie Militaíre, Palerme, 56 pp. 10.5962/bhl.title.6135

[B39] Randall JW (1840) Catalogue of the Crustacea brought by Thomas Nutall and J.K. Townsend, from the West Coast of North America and the Sandwich Islands, with descriptions of such species as are apparently new, among which are included several species of different localities, previously existing in the collection of the Academy. Journal of the Academy of Natural Sciences of Philadelphia 8: 106–147. [pls 3–7] 10.5962/bhl.title.119921

[B40] Stebbing TRR (1914) South African Crustacea. Part VII of S.A. Crustacea, for the Marine Investigations in South Africa. Annals of the South African Museum 15: 1–55. [pls 1–12] 10.5962/bhl.part.22194

[B41] von Sternberg R (1997) Phylogenetic and systematic position of the PenaeussubgenusLitopenaeus (Decapoda: Penaeidae). Revistra de Biologia Tropical 44(3)–45(1): 441–451.

[B42] von Sternberg R Motoh H (1995) Notes on the phylogeny of the American *Penaeus* shrimps (Decapoda: Dendrobranchiata: Penaeidae). Crustacean Research 24(0): 146–156. 10.18353/crustacea.24.0_146

[B43] Stimpson W (1871) Notes on North American Crustacea, in the museum of the Smithsonian Institution. No. III. Annals of the Lyceum of Natural History of New York 10: 119–163. [preprint issued in1871, paper in bound volume in 1874 with page numbers 92–136]

[B44] Streets TH (1871) Catalogue of Crustacea from the Isthmus of Panama, collected by J.A. McNeil. Proceedings. Academy of Natural Sciences of Philadelphia 1871: 238–243.

[B45] Tavares C Gusmão J (2016) Description of a new Penaeidae (Decapoda: Dendrobranchiata) species, *Farfantepenaeusisabelae* sp. nov. Zootaxa 4171(3): 505–516. 10.11646/zootaxa.4171.3.6 27701214

[B46] Timm L Browder JA Simon S Jackson TL Zink IC Bracken-Grissom HD (2019) A tree money grows on: the first inclusive molecular phylogeny of the economically important pink shrimp (Decapoda: *Farfantepenaeus*) reveals cryptic diversity. Invertebrate Systematics 33: 488–500. 10.1071/IS18044

[B47] Tirmizi NM (1971) *Marsupenaeus*, a new subgenus of *Penaeus* Fabricius, 1798 (Decapoda, Natantia). Pakistan Journal of Zoology 3: 193–194.

[B48] Tsoi KH Ma KY Fennessy ST Chu KH Chan TY (2014) Verification of the cryptic species *Penaeuspulchricaudatus* in the commercially important kuruma shrimp *P.japonicus* (Decapoda: Penaeidae) using molecular taxonomy. Invertebrate Systematics 28(5): 476–490. 10.1071/IS14001

[B49] Yang CH Ma KY Chu KH Chan TY (2023) Making sense of the taxonomy of the most commercially important shrimps *Penaeus* Fabricius, 1798 s. l. (Crustacea: Decapoda: Penaeidae), a way forward. Aquaculture (Amsterdam, Netherlands) 563: 1–10. 10.1016/j.aquaculture.2022.738955

[B50] Yu HP Chan TY (1986) The Illustrated Penaeoid Prawns of Taiwan. Southern Materials Center, Taipei, 183 pp.

